# A comparative study of femtosecond pulsed and continuous wave lasers on physiological responses through activation of phytochromes in seeds

**DOI:** 10.1038/s41598-025-11183-8

**Published:** 2025-07-23

**Authors:** Csenger Márk Szabó, Botond Bán, Borbála Sinka, Bálint Tóth, Barnabás Gilicze, Imre Seres, János Bohus, Attila Ébert, Péter Borbély, Zsolt Gulyás, Gábor Galiba, Eva Darko, Miklós Hovári, Béla Hopp, Csaba Péter, Károly Mogyorósi, András Viczián

**Affiliations:** 1 The Extreme Light Infrastructure ERIC | ALPS Facility, Wolfgang Sandner u. 3, Szeged, H-6728 Hungary; 2 Department of Optics and Quantum Electronics, University of Szeged, Dóm tér 9, Szeged, H-6720 Hungary; 3 Agricultural Institute, HUN-REN Centre for Agricultural Research, Brunszvik u. 2, Martonvásár, H-2462 Hungary; 4 Department of Agronomy, Hungarian University of Agricultural and Life Sciences, Georgikon Campus, Deák Ferenc u. 16, Keszthely, H-8360 Hungary; 5 Plant Stress and Phenomics Group, Institute of Plant Biology, HUN-REN Biological Research Centre, Temesvári krt. 62, Szeged, H-6726 Hungary; 6 Laboratory of Photo and Chronobiology, Institute of Plant Biology, HUN-REN Biological Research Centre, Temesvári krt. 62, Szeged, H-6726 Hungary; 7 Doctoral School of Biology, Faculty of Sciences and Informatics, University of Szeged, Közép fasor 52, Szeged, H-6726 Hungary

**Keywords:** Femtosecond pulsed laser, Germination, Plant phenotyping, Photomorphogenesis, Phytochrome B, Ultrafast photoswitch, Ultrashort light pulse, Light responses, Ultrafast lasers

## Abstract

Red light activates phytochrome photoreceptors, which mediate such key developmental steps as germination and seedling photomorphogenesis in *Arabidopsis thaliana*. To examine the details of these responses, we developed a novel experimental system and demonstrated that brief, high-intensity light pulses can elicit sustained physiological responses. We observed that the seeds responded to the femtosecond laser light pulses, but with lower sensitivity compared with continuous light sources having the same average fluence. We concluded that (i) phytochrome B photoreceptors within imbibed seeds efficiently absorb red and far-red photons from pulsed femtosecond laser pulses, with absorption occurring during approximately 10 orders of magnitude shorter amount of time than with conventional light sources; (ii) these treatments did not induce adverse effects during later plant development; and (iii) the effect of ultrashort light pulses *in planta* coincides with phytochrome photoconversion characteristics described during in vitro studies. Our findings demonstrate that seed germination and photomorphogenic development can be effectively triggered by light, regardless of whether it is delivered continuously or within extremely brief pulses. This research expands the potential applications of femtosecond laser technology and demonstrates the feasibility of investigating the effects of ultrafast physical phenomena on biological processes in vivo using diverse biological readouts.

**Supplementary Information:**

The online version contains supplementary material available at 10.1038/s41598-025-11183-8.

## Introduction

Light is an important environmental factor that influences almost all developmental steps of plants. The perception of photons, and thus the control of light signaling pathways, is governed by light sensitive photoreceptor molecules. Their concerted action results in the manifestation of light-mediated growth and developmental patterns, including germination and photomorphogenesis^1,2^. Quantification of these responses can be used to monitor photoreceptor signaling *in planta*^3^.

Small seeds (e.g., *Arabidopsis thaliana*, lettuce, tomato, tobacco, several weed species, etc.) have limited nutrient reserves, therefore light provides essential cues about their depth in the soil, helping determine whether the seedlings can successfully emerge and grow into mature plants^4–9^. Arabidopsis show limited germination in darkness, but light induce the process by activating the phytochrome (PHY) photoreceptors^10–12^which are the sensors of red (R, ʎ_max_~660 nm) and far-red (FR, ʎ_max_~730 nm) light. Arabidopsis has five phytochromes (phyA-E); among them, phyB is the dominant photoreceptor mediating most R light-driven plant responses including germination^12–15^ and seedling photomorphogenesis^16–19^. Phytochromes have a heme-derived linear tetrapyrrole chromophore attached, which provides light sensitivity to the molecule. Phytochromes are synthesized in their inactive (Pr) form, and upon R light perception, they are converted to the biologically active Pfr conformer. This conformational change is necessary for phytochrome-driven light signaling. Pfr can be reverted back to Pr by FR irradiation or through spontaneous thermal reversion^20–22^.

The temporal dynamics of the phytochrome Pr-Pfr conformation changes have been extensively investigated, but most studies have focused on the bacterial counterparts of plant phytochromes^21,23,24^. Additionally, most of the data were generated by in vitro approaches using isolated phytochrome-containing protein extracts^24–28^. However, some results suggest that these observations may also apply to plant phytochromes^29,30^. These results show that, similarly to other photoreceptors, the earliest phytochrome light sensing photochemical events occcur on the femto/picosecond timescale, whereas the subsequent protein conformational changes on the millisecond timescale after the light illumination^30–32^. The photoconversion processes, which span a diverse temporal range, have been examined in both plant and bacterial phytochromes. These processes unfold over similar durations and involve comparable intermediate states, despite variations in their chromophores and attachment sites^33,34^. These light-driven transformations initiate with the Z-to-E isomerization of the C-D methine bridge double bond, resulting in the forming of Lumi-R intermediate^34,35^. After the excitation of PHY A (in vitro) by 665 nm red light, the lowest excited Pr singlet state (S_1_) is formed at around 100 fs and accompanied by strong vibrational oscillations lasting up to 1 ps. By approximately 1–2 ps, the excited state begins to relax. After overcoming a barrier on the S_1_ excited state surface the first ground-state intermediate, Lumi-R appears (25–30 ps)^30,36^ and exhibits a highly strained chromophore configuration^34,35^. The Lumi-R formation occurs with a yield of approximately 10–15%, while the rest of the excited Pr population relaxes to ground state within 3–6 ps^27,32,37^. Following a subsequent thermal relaxation of Lumi-R, the Meta-Ra intermediate emerges on the µs timescale through relaxations of the C-D methine bridge double bond. This is followed by the formation of Meta-RC (ms scale), characterized by rearrangements of pyrrole rings B and C within the protein pocket. These sequential steps involve coordinated adjustments of both the chromophore and its immediate protein surroundings^34–36^. For the Meta-R intermediates, a recovery pathway also exists, leading back to ground state Pr formation^34,38^. This recovery pathway further lowers the overall Pfr yield. Ultimately, conversion from Meta-Ra to the Pfr state (ms to s timescale) involves substantial structural changes in the protein scaffold, including proton movement in the chromophore pocket and an α-helix transformation in the tongue region of the PHY domain, which leads to the modulation of the downstream signal transduction^34,35^. As soon as a sufficient amount of generated Pfr reached, the germination response is induced, hours before the first visible changes on the seed^13^. This light requirement can be fulfilled even in minutes, depending on species^39,40^. Charactheristics of Pr photochemistry preditct that the initiation of sufficient Pfr formation may occur in picosecond-to-millisecond timescale – including Pr (re)excitation in femtoseconds – thus determining the light-induced germination response under red light. The time resolution of Pfr photoconversion shows similar pattern to that of Pr^30,37^.

Beyond in vitro experimental approaches, in vivo plant studies have revealed that short light pulses can activate phytochrome-mediated signaling^12,38,41^. Extremely short pulses can be generated using lasers, but it should be noted that, in photobiology studies the phrase “light pulse” refers to short-term irradiation in the millisecond–minute range, whereas in laser-related literature, photons arriving in a “pulse” are delivered in time periods of a few nanoseconds to femtoseconds. Our knowledge of phytochrome function under ultrashort pulse irradiation, when light is delivered in sub-picosecond flashes, is rather limited. By examining phytochrome function, we can gain insights into how effectively these special irradiation regimes trigger conformational changes and influence photomorphogenic development. Germination is an excellent phenotypic trait for examining the physiological effects of these short pulses. It is well known that germination can be triggered by short R and inhibited by FR irradiation periods on the minute scale, and this response is mediated by phytochromes^12,14,41^. The physiological effects of shorther light pulses have also been investigated. Millisecond-long R light flashes effectively induce germination when delivered frequently, and their effect can be reverted by FR flashes^42^. Furthermore, the application of nanosecond red laser flashes can also induce germination effectively, making lasers valuable tools to examine plant responses induced by short light pulses^38^. However, femtosecond laser pulses, which are much shorter in duration, have not been applied to plants, and the effects of laser irradiation on plant responses in phytochrome mutants have not been studied, leaving the direct role of these photoreceptors in these processes unclear.

The physiological effects of continuous wave (cw) laser irradiation on plants were investigated more than five decades ago^43^and a substantial collection of reports has accumulated since then (reviewed recently by Dudareva^44^). These studies focuses mostly on seed or plant improvement, since the application of chemical methods to improve seed characteristics becomes increasingly restricted due to legal regulations, the need to explore new technologies for pre-sowing treatments has grown^44,45^. Although the use of laser irradiation for seed treatment is a rather narrow field of laser applications, the number of relevant publications in Scopus-indexed journals has shown a growing trend over the past five years. These studies include irradiation protocols using various laser setups applied to seeds from diverse plant species at different developmental stages, which makes comparing the results difficult. Whereas plant responses to cw lasers have been extensively investigated, our knowledge of *in planta* responses induced by short laser flashes, especially in the pico-femtosecond range, remains scarce. Additionally, the introduction of well-defined methods is required to better understand the underlying mechanisms of laser-induced physiological changes.

We set two major aims for our study (i) to develop a precise irradiation sytem that allows the comparison of different light sources, including femtosecond lasers, thereby paving the way for their applications in further studies, and (ii) to characterize the physiological effects of ultrashort (~ 100 fs) red laser pulses and to compare them to those of short (< 10 s) pulses applied to Arabidopsis seeds. The application of these femtosecond pulses enables the systematic, time-dependent characterization of reversible biological responses, exhibiting kinetics resolvable on the µs-ms timescale. This approach allows for the comparison of biological effects induced by different light sources using standardized irradiation protocols, with these effects serving as a quantifiable metric to assess photoreceptor function. We found that phytochromes, located in the seeds, can perceive ultrashort laser pulses and mediate corresponding physiological responses. In conclusion, this work illustrates how brief physical changes in the environment can induce profound and long-lasting effects on plant growth and development, which can be monitored in vivo by quantifying phenotypic traits.

## Materials and methods

### Plant materials

We used *Arabidopsis thaliana* Landsberg *erecta* (L*er*) ecotype (Nottingham Arabidopsis Stock Centre, UK) as a wild-type (WT). Previously characterized higher-order phytochrome mutants, derived from L*er* ecotype and obtained from their original investigators, were employed in this study. They are abbreviated as follows: abcde: (*phyA-201phyB-1phyC-1phyD-1phyE-1*)^46^; aBcde: (*phyA-201phyC-1phyD-1phyE-1*)^47^.

**Fig. 1:**
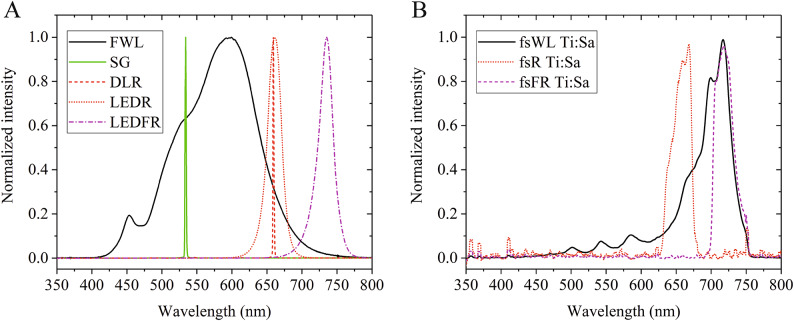
Emission spectra of the applied light sources. (**A**) Continuous wave light sources: white light halogen lamp in the incubator (FWL, 425–750 nm), the safe green laser (SG, 532 nm), red diode laser (DLR, 660 nm), red LED (LEDR, 600–700 nm) and far-red LED (LEDFR, 670–780 nm). (**B**) Femtosecond pulsed laser sources as the fsWL (450–750 nm with FES0750 short pass filter), fsR (620–680 nm with FB650-40 band pass and FES0750 short pass filter) and fsFR (690–750 nm with FEL700 long pass and FES0750 short pass filter) were generated using the Titanium: Sapphire (Ti: Sa) laser.

**Fig. 2:**
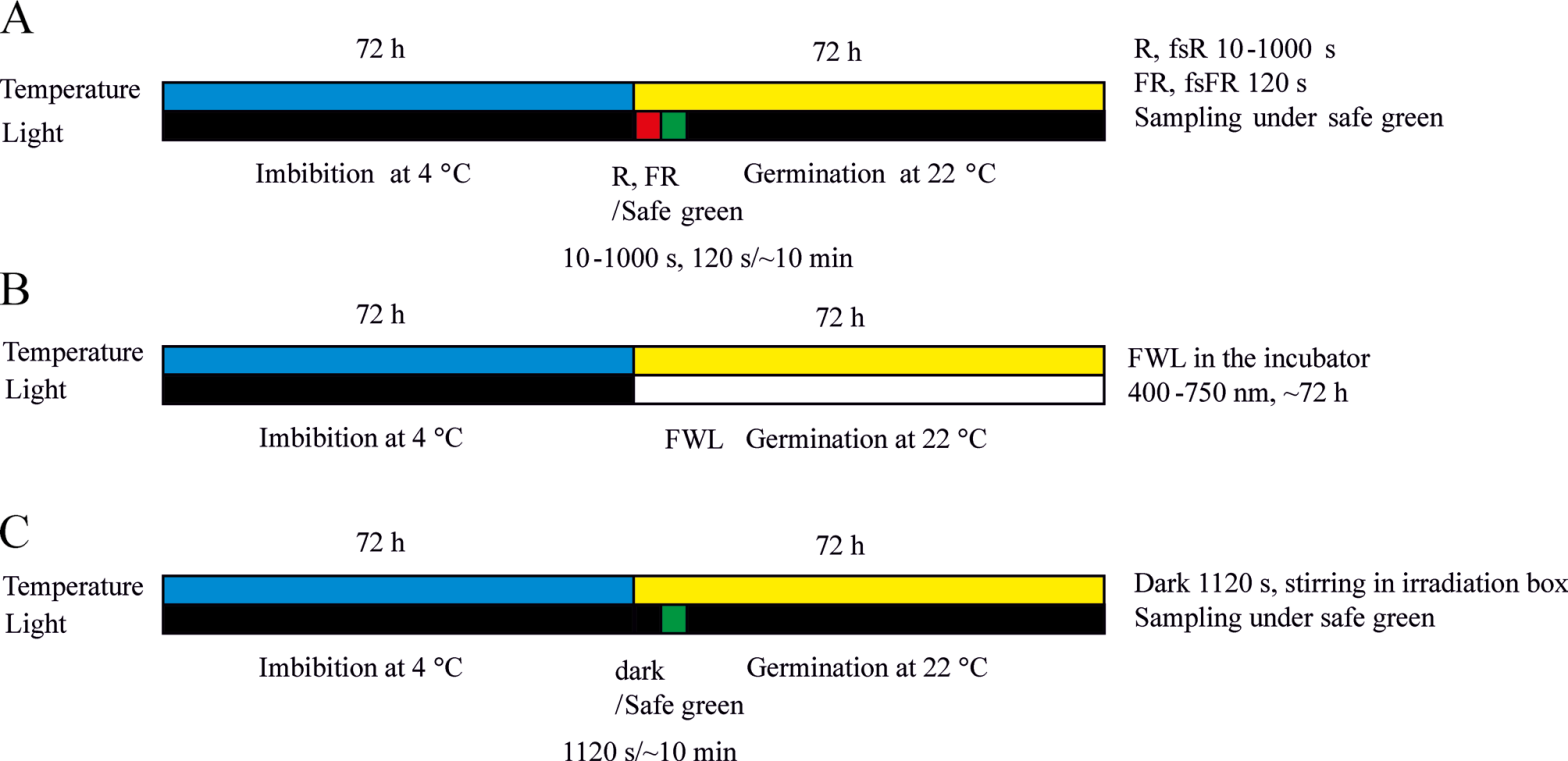
Seed germination protocol indicating the different light and temperature treatments. Schematic illustration of the light irradiation and incubation of seeds at different temperatures during experimentation. Seeds were imbibed in water at 4 °C then irradiated and germinated at 22 °C. The irradiation protocols were as follows: (**A**) The following short (< 1000 s) irradiation with cw LEDR or LEDFR (incoherent 660 nm R or 725 nm FR light, respectively); femtosecond red (fsR, 630–670 nm) or femtosecond far red (fsFR, 700–750 nm) pulsed coherent laser light at the Ti: Sa or HR1 laser. Subsequently, the samples were processed under safe green light and incubated in the dark for 72 h at 22 °C. (**B**) Full white light (FWL) irradiation (425–750 nm) for 72 h after imbibition. (**C**) Dark control with no irradiation; all sample handling was done under diffuse safe green light.

Typically, approximately 300 seeds (weighing 8.0 ± 0.2 mg) seeds were measured in a glass vial for each treatment group at room temperature and normal room light conditions. A 50 seed/mL seed suspension was created by adding 6.0 mL of high purity water (VWR PuranityTU6 UV/UF+) to each vial in a dark room illuminated with safe green light (532 nm, Fig. 1). The vials were all sealed with parafilm and wrapped in multiple layers of aluminum foil to prevent any light penetration and were stored at 4 °C for 72 h (Fig. 2). When the imbibition was completed, the samples, covered with layers of aluminium foil, were taken to the irradiation lab and kept at room temperature for 2 h in order to adjust their core temperature to the environment before subsequent light treatments.

### Irradiation of the seeds

Every irradiation treatment took place in cleanroom areas, in compliance with the ISO 7 standard at the ELI ALPS Research Institute, ensuring consistent conditions (23.0 ± 0.5 °C, RH 35%). When the samples were irradiated, only safe green light (CPS532 laser at a power of 4.5 mW; Thorlabs, Newton, NJ, USA) was used in the room (Fig. 1A). Similarly, water was added to the seeds for imbibition in a dark room under safe green light (Fig. 2). The seeds were not subjected to direct green light; only diffuse and scattered light was used to ensure visibility during sample handling. This irradiation did not affect any physiological responses we observed.

The vials, containing seeds imbibed in water, were opened immediately before the irradiation from the top and bottom of the vessel. To ensure the comparability of the experiments, we applied the same key parameters in every irradiation setup. The diameter of the beam was set to 9 mm at the neck of the vial containing the seeds, expanding by about 10% at the bottom of the sample holder. All calculations regarding the fluence rate were done using these settings. Homogeneous light exposure in the aqueous seed suspension was provided by stirring with a magnetic stir bar at a constant rate, which moved each seed through slightly different voxels of the irradiated volume. During light irradiation, the seeds were protected from any unwanted light (e.g. trace amount of scattered light from any optical elements) by wrapping the wall of the vial with aluminium foil. The duration of irradiation was measured with 1-second and 10-millisecond accuracy when the duration of the treatment was in the minute range or in the 1 s range, respectively. Dark controls were treated the same manner as the irradiated samples, with the seed suspension stirred, but without the specific irradiation treatment. The temperature of the samples was kept at 23 ± 1 °C.

### Germination assays and measurement of hypocotyl length

After irradiation, the seeds were transferred into 96-well microplates (Thermo Fisher Scientific, Waltham, USA) with a micropipette (100 µL seed suspension, 5–7 seeds in each well) under ambient safe green light. The plates were sealed using parafilm to prevent water evaporation and were kept in complete darkness in a black metal box sealed with a non-transparent tape, except for the Full White Light (FWL) treatment, until evaluation. The plates were maintained at a constant 22.0 ± 0.1 °C temperature provided by a Lab-Therm LT-X (Kuhner, Basel, Switzerland) incubator for 72 h (Fig. 2).

The germinating seeds were imaged using an EnSight plate reader (PerkinElmer, Waltham, MA, USA) according to the manufacturer’s instructions. The two wavelengths used for imaging were 385 nm (30 ms exposure time, excitation power: 100%) and 735 nm (4 ms, excitation power: 5%) for autofluorescence and bright-field imaging respectively (Figure S2). The scanning procedure took about two minutes, thus all samples were scanned under constant environmental conditions (temperature, light). The number of germinating seeds was determined with the ImageJ software (version 1.52a)^48^. A seed was considered germinating when the tip of the embryo root tip became visible.

For the determination of hypocotyl length, at least 15 seedlings from each light treatment were placed in a 3% agar medium containing 1% charcoal and scanned using an Epson Perfection V30 (Epson, Nagano, Japan) scanner at 800 dpi resolution. Hypocotyl length was measured using the ImageJ software. Germination and hypocotyl data were analyzed and graphed using Origin 9.4 (OriginLab, Northampton, MA, USA) and GraphPad Prism 10 (Boston, MA, USA) softwares.

### Adult plant phenotyping

About 300 seeds were irradiated as described above and were germinated on soil at two sites, as follows: Site 1: Biological Research Centre, Szeged, Hungary (Phytoclima, Aralab, Rio de Mouro, Portugal) and Site 2: Centre for Agricultural Research, Martonvásár, Hungary (ST 500P SMART PRO, POL-EKO-APARATURA SP.J., Wodzisław Śląski, Poland). The controlled plant chambers provided 8 h white light (100 µmol m^–2^s^–1^) / 16 h dark photocycles at 22 °C at both locations.

Adult plant phenotyping was done using PSI-Compact (Site 1) and PSI-Plantscreen (Site 2) plant phenotyping systems (Photon System Instruments, Drasov, Czech Republic). Morphological imaging was performed with the system’s top mounted RGB camera (13 Mpix, CMOS-sensor). Fluorescence imaging was carried out, after 20 min of dark adaptation of the plants, using the station’s own FluorCam FC-800MF Pulse Amplitude Modulated (PAM) system, equipped with a TOMI-2 High Resolution CCD camera (1360 × 1024 px resolution). Hyperspectral imaging was carried out with a CMOS sensor (1920 × 1000 px resolution, 12-bit depth, wavelength range of 380–900 nm) and a halogen lamp with a 1860 K color temperature to obtain calibration images. All morphometric and photosynthetic parameters were calculated according to the manufacturer’s instructions.

### Characterization of the light sources

The spectra of the applied light sources were measured using an optical fiber-coupled Ocean Optics USB2000 + spectrometer (Ocean Optics, Orlando, FL, USA) at the sample position directly before the experiments. The collimated beams were focused onto an optical fiber with a reflection collimator. The integration time was typically 100 ms, and 10 spectra were averaged.

**Fig. 3:**
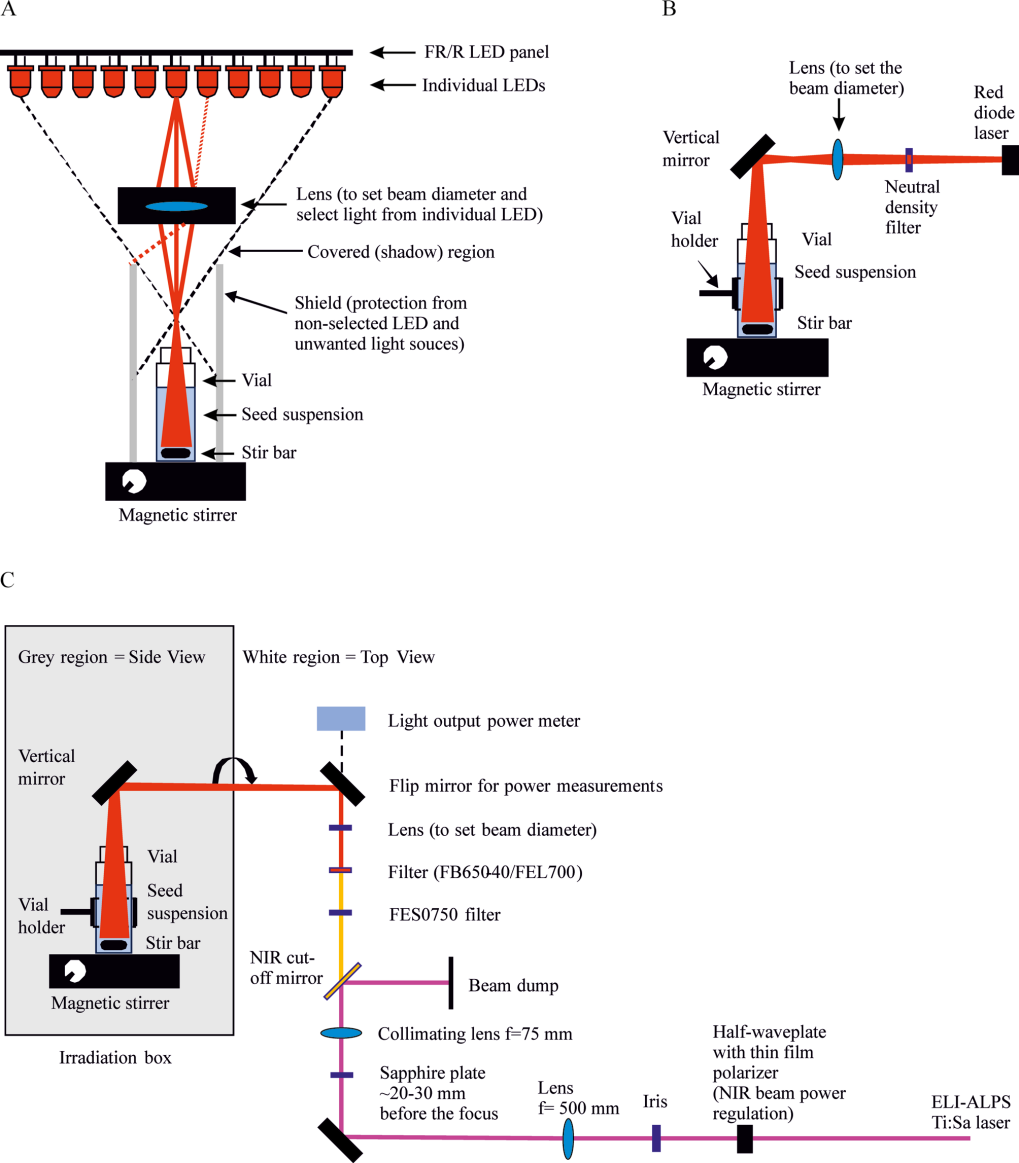
The schematic drawing of the irradiation setup. (**A**) LED irradiation setup with LEDR or LEDFR panels collimating a single LED light with a plano-convex lens to adjust the beam diameter and a black metal lens tube for proper shadowing from other LEDs in the panel and magnetic stirring; (**B**) DLR irradiation setup adjusting the beam diameter with two lenses and directing the beam vertically downward into the sample vial with a periscope mirror. (**C**) fsWL (450–750 nm, with FES0750 short pass filter), fsR (620–680 nm, with FB650-40 band pass and FES0750 short pass filters) and fsFR (690–750 nm, with FEL700 long pass and FES0750 short pass filters) irradiation setup at the Titan: Sapphire (Ti: Sa, 782 nm, 1 kHz, ~ 100 fs) or HR1 (1030 nm, 100 kHz, ~ 30 fs) laser generated on a sapphire plate focusing the NIR beam on the crystal and collimated to the suitable beam diameter.

We used custom-built panels^49^ containing red or far-red light emitting diodes (LEDR for 660 nm and LEDFR for 725 nm irradiation, respectively) as incoherent references (Table 1; Fig. 1A). The applied experimental design and the accurate determination of the fluence rate required the projection of light emitted from a single diode. The desired diameter at the top of the vial was set to 9 mm with a plano-convex lens (Fig. 3A). The power output was used to set the fluence rate.

**Table 1:** Coherence length of the applied light sources.

Light source	Wavelength (nm)	Coherence length	Light source type	Duration of the irradiation	Applied fluence (µmol/m^2^)
White light lamp (FWL)	425–750	incoherent	cw	3 days	6625000
LEDR	600–700	incoherent	cw	10–1000 s	85-8500
LEDFR	670–780	incoherent	cw	120 s	11400
Safe green laser	532	< several mm	cw	NA	NA
Red diode laser (DLR)	660	< several mm	cw	10–1000 s	85-8500
fsWL Ti: Sa laser	450–750	5.2 μm	pulsed	NA	NA
fsR Ti: Sa laser	620–680	6.8 μm	pulsed	10–1000 s	85-8500
fsFR Ti: Sa laser	700–750	7.4 μm	pulsed	120 s	8600
fsWL HR1 laser	400–750	1.7 μm	pulsed	NA	NA
fsR HR1 laser	620–680	4.4 μm	pulsed	10–1000 s	23-2300

NA: not applicable; cw: continuous wave.

A red diode laser (DLR, ML101J27; Mitsubishi, Tokyo, Japan) was used with a controller (Thorlabs, LDC 220 C) as shown in Fig. 3B. The spectrum of the DLR light has a maximum at 660 nm (nearly monochromatic, Fig. 1A). The expanding beam from the diode was focused on the vial using a lens. The accuracy of the timing during short-term irradiation (0.1–10 s) was ensured by an automatic shutter (Thorlabs, SH05R/M).

The 1 kHz femtosecond laser irradiation was primarily performed using a Titanium Sapphire (Ti: Sa) laser (Arco, Amplitude, Bordeaux, France) producing 100 fs pulse duration at a maximum pulse energy of 4 mJ/pulse. The femtosecond pulsed white light (fsWL Ti: Sa) was generated when the primary near infrared (NIR) 782 nm beam of the Ti: Sa femtosecond laser (with 150 mW average power) was focused onto a 1 mm thick sapphire window. The near infrared portion of the white light was separated via a NIR cutoff mirror (Fig. 3C). The remaining NIR radiation was filtered out by a FES0750 short-pass filter (Thorlabs), therefore the utilized femtosecond laser white light had a spectrum of 475 nm to 750 nm (Fig. 1B). Femtosecond pulsed red light (fsR Ti: Sa) was obtained from the white femtosecond laser beam by using the combination of a FB650-40 bandpass filter (Thorlabs), which allows the transmission of the 630–670 nm red light, and the FES0750 short-pass filter to ensure that any potentially remaining near infrared light was completely removed from the primary beam. The center wavelength (650 nm) is very close to the emission spectrum maxima of the LEDR and DLR light sources (660 nm) (Fig. 1B). The average power of fsR light was 100 µW in these experiments. Femtosecond pulsed FR light (fsFR Ti: Sa) was separated from the white femtosecond laser beam using the combination of a FEL700 long-pass filter and the FES0750 short-pass filter (Thorlabs), resulting in a 700–750 nm spectral range (Fig. 1B). The fsFR light was used at 1000 µW average power.

The 100 kHz femtosecond pulsed HR1 laser produced 1030 nm NIR radiation, centered at the source wavelength^50^. The laser consists of an ytterbium fiber chirped-pulse amplifier and delivers a 25 W-30 fs output, with the power that can be independently controlled from the main beam. During the experiment, the beam was characterized by a 100 µJ pulse energy (10 W average power), a spectral wavelength range of 980–1080 nm, and an approximately transform-limited 30 fs pulse duration. The applied average power of the fsR HR1 beam, separated by the bandpass filter, was 27 µW, using the same filters as described for selecting the fsR Ti: Sa light (Figure S1)^51^.

To determine the absolute endpoint for the light treatments (maximum germination rate), we used continuous white light (FWL, Fig. 2), provided by the built-in lamp of the Lab-Therm LT-X incubator, which had a spectrum spanning from 425 to 750 nm (Fig. 1A).

Besides the applied spectrum and pulse length, the coherence length is another fundamental difference between the applied light sources. The FWL and the LED (LEDR/LEDFR) sources provide fully incoherent radiation (Table 1). In case of the safe green and DLR lasers, the coherence length is in the range of several mm due to the high number of roundtrips in the resonator. The third group of applied light sources are the fs laser-based WL sources and their filtered components. Both the Ti: Sa and the HR1 lasers produce transform-limited pulses (where the product of bandwidth and pulse duration is minimized). If multifilamentation is avoided during WL generation, the generated WL is also coherent. We calculated the coherence length using this Eqs.^52,53^:

$$\:{L}_{coh}=\frac{2ln2}{\pi\:}\frac{{\lambda\:}^{2}}{\varDelta\:\lambda\:}$$

The number of photons used for each treatment was determined in units of µmol m^– 2^ s^– 1^. To calculate the fluence rate for each treatment, the diameter of the beam of each light source was fixed at 9 mm. The power output was then measured at the upper rim of the vial (Fig. 3) using a PH100-Si-HA-OD1-D0 (gentec-EO, Quebec, QC, Canada) power meter. The spectrum and the diameter of the laser beam were set before the experiments and the final output power was carefully measured frequently between irradiations to minimize any errors arising from potential instability of the generated fsWL pulsed beam. The required fluence rate and fluence was calculated using the following correlations where *ν* is the frequency of the light, *c* is the speed of light, *λ* is the wavelength of light, *h* is the Planck constant, *E*_*p*_ is the energy of one photon, *r* is the radius of the beam, *A* is the area of the irradiated surface, *N*_*p*_ is the number of photons and *P* is the beam power:

$$\:\nu\:=\frac{c}{\lambda\:}$$

$$\:{E}_{p}=h\nu\:$$

$$\:A={r}^{2}\pi\:$$

$$\:{N}_{P}=\frac{Pt}{{E}_{p}}$$

$$\:\:\varPhi\:=\frac{{N}_{p}}{tA}$$

The *photon flux* (*Φ*) was expressed as the number of photons crossing the unit area in unit time in metric units using the following formula based on the measured parameters:

$$\:\varPhi\:\left[\frac{photon}{{m}^{2}s}\right]=\frac{P}{h\nu\:}\frac{1}{A}=\frac{P}{h\frac{c}{\lambda\:}{r}^{2}\pi\:}=\frac{P\lambda\:}{hc{r}^{2}\pi\:}$$

Converting number of photons to moles of photons, the *fluence rate* (*Φ*_*f*_) is:

$$\:{\varPhi\:}_{f}\left[\frac{mol}{{m}^{2}s}\right]=\frac{P\lambda\:}{hc{r}^{2}\pi\:}\frac{1}{{N}_{A}}$$

Multiplying the fluence rate by the time of irradiation gives us the total molar number of photons crossing through the unit area throughout the treatment as *fluence (F*):

$$\:F\left[\frac{mol}{{m}^{2}}\right]={\varPhi\:}_{f}t$$

We used later the practical units for fluence rate (µmol m^– 2^s^– 1^) and fluence (µmol m^– 2^).

## Results

### Reducing the time of saturation with high fluence rate DLR irradiation

**Fig. 4:**
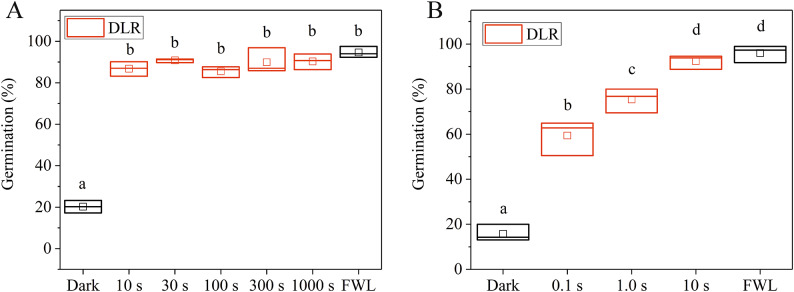
Saturation of germination with red diode laser irradiation. Wild-type Arabidopsis seeds were irradiated with a cw red diode laser (DLR, 660 nm, 6000 µmol m^– 2^s^– 1^) for longer (**A**, 10–1000 s) or shorter (**B**, 0.1–10 s) irradiation time periods and germinated for 72 h in the dark at 22 °C or kept under constant white light (FWL). The percentage of germination was determined in three replicates with the total number of seeds per treatment group *N* > 300 and *N* > 65 per replicates in a subgroup (mean ± SE; *n* = 3). The line bisecting each box represents the median value; the lower and upper edge of the box indicate the minimum and the maximum values, respectively. The empty square within each box indicate the mean value. The letters a–d indicate significant differences in the means (*p* < 0.05 by ANOVA followed by a Tukey Test).

One of our main goals was to determine the minimum irradiation time required to initiate the germination of imbibed seeds by activating photoreceptor-driven molecular pathways. To achieve this, we developed a simple experimental setup that allowed us to examine the effect of short-term irradiation on the germination of seeds (Fig. 3). First, we applied short (10–1000 s), yet intense (70 mW, 660 nm, 6000 µmol m^– 2^s^– 1^) DLR irradiation pulses (Fig. 4A). The results show that even a short irradiation of 10 s can nearly maximize the germination rate (compared to FWL treatment the difference is insignificant). Next, we applied shorter irradiation times and found that even a 0.1 s pulse induces a significantly higher germination rate compared with the dark control. Increasing the duration of the R light pulse resulted in a higher germination rate, reaching saturation after a 10 s pulse (Fig. 4B). These results suggest that light illumination remains effective in inducing germination, even when its duration is significantly reduced from minutes to seconds as long as a high fluence rate is provided.

### Fluence dependence of short pulses generated by different light sources

**Fig. 5:**
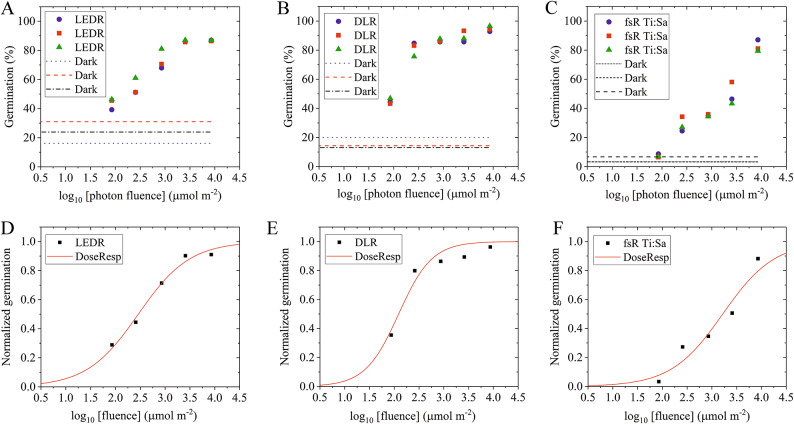
Fluence dependent germination induced by different red-light sources. Arabidopsis wild-type seeds were irradiated with R light at 8.5 µmol m^– 2^ s^– 1^ fluence rate (100 µW average power), for 0, 10, 30, 100, 300 and 1000 s resulting in 85–8500 µmol m^– 2^ total fluence during the treatments using the following light sources: (**A**) Red LED irradiation (LEDR, 600–700 nm). (**B**) Red diode laser irradiation (DLR, 660 nm). (**C**) Femtosecond red pulsed light irradiation generated with the Ti: Sa laser (fsR, 620–680 nm) (**D**) LEDR, (**E**) DLR and (**F**) fsR Ti: Sa, based on the average values from panels A, B and C, respectively. Sigmoid curves were calculated using the dose response function of the Origin software based on the normalized data, setting the minimum values (dark) 0, and the maximum (Full White Light) 1.0. Each measurement was performed in three replicates (blue, red, green symbols) with the total number of seeds per treatment group being *N* > 200 and *N* > 50 per replicates in a subgroup.

To better characterize how germination depends on the quality of the initiating light pulse, we used three different red-light sources and applied the same photon fluence to imbibed seeds and determined their germination rate (Fig. 2). First, we tested a conventional LED light source (Figs. 1A and 3A). Using a constant fluence rate (8.5 µmol m^– 2^ s^– 1^), we selected the duration of irradiation between 10 and 1000 s, determined the germination rate and plotted it according to the received fluence (shown on a logarithmic scale in Fig. 5A). The results show a monotonic increase in the germination rate with increasing fluence, reaching saturation (87% within 10% of FWL level) at 3.5 log_10_(fluence) (Fig. 5A). Fitting a sigmoid curve on the normalized data reveals an EC50 (half maximal effective concentration) at 2.46 log_10_(fluence) indicating that a fluence of 290 µmol m^– 2^ resulted in a 50% germination effect compared to the dark and FWL controls (Fig. 5D).

Second, we irradiated the seeds with another cw light source, the red diode laser (DLR, Figs. 1A and 3B), that produces coherent irradiation. We applied the same fluence as for LEDR. A monotonic increase in germination rate can be observed, reaching a saturating fluence value of 8500 µmol m^– 2^ (95%, not significantly different from the FWL of 96%) at 3.9 log_10_(fluence). Fitting a sigmoid curve on the normalized data reveals an EC50 at 2.09 log_10_(fluence), indicating that a fluence of 123 µmol m^– 2^ resulted in a 50% germination effect between the dark and FWL conditions (Fig. 5B and E).

In the third experiment we used the coherent, pulsed fsR Ti: Sa femtosecond laser light source (Fig. 1B), applying the same experimental settings as in the previous two experiments (Fig. 3C). We observed a fluence-dependent monotonic increase in germination (Fig. 5C), but the characteristics of the curve fitted to the experimental data were significantly different from the previous two curves (Fig. 5D and E), shifting towards higher fluences. A nearly saturating level of fluence was reached at 8500 µmol m^– 2^ (83% germination rate compared with 94.4% of FWL). Fitting a sigmoid curve to the data yields an EC50 of 3.22 log_10_(fluence), which means that a fluence of 1660 µmol m^– 2^ resulted in a 50% response between dark and FWL conditions (Fig. 5F). A similar EC50 value was obtained using the fsR HR1 laser, resulting in an EC50 of 3.33 log_10_(fluence), meaning that a fluence of 2140 µmol m^– 2^ was sufficient to achieve 50% germination (Figure S3). These results indicate that significantly higher fluences of femtosecond pulsed laser irradiation are required to induce the same level of germination achieved using lower fluences of cw light sources.

### Red/far-red reversibility induced by femtosecond laser pulses

**Fig. 6:**
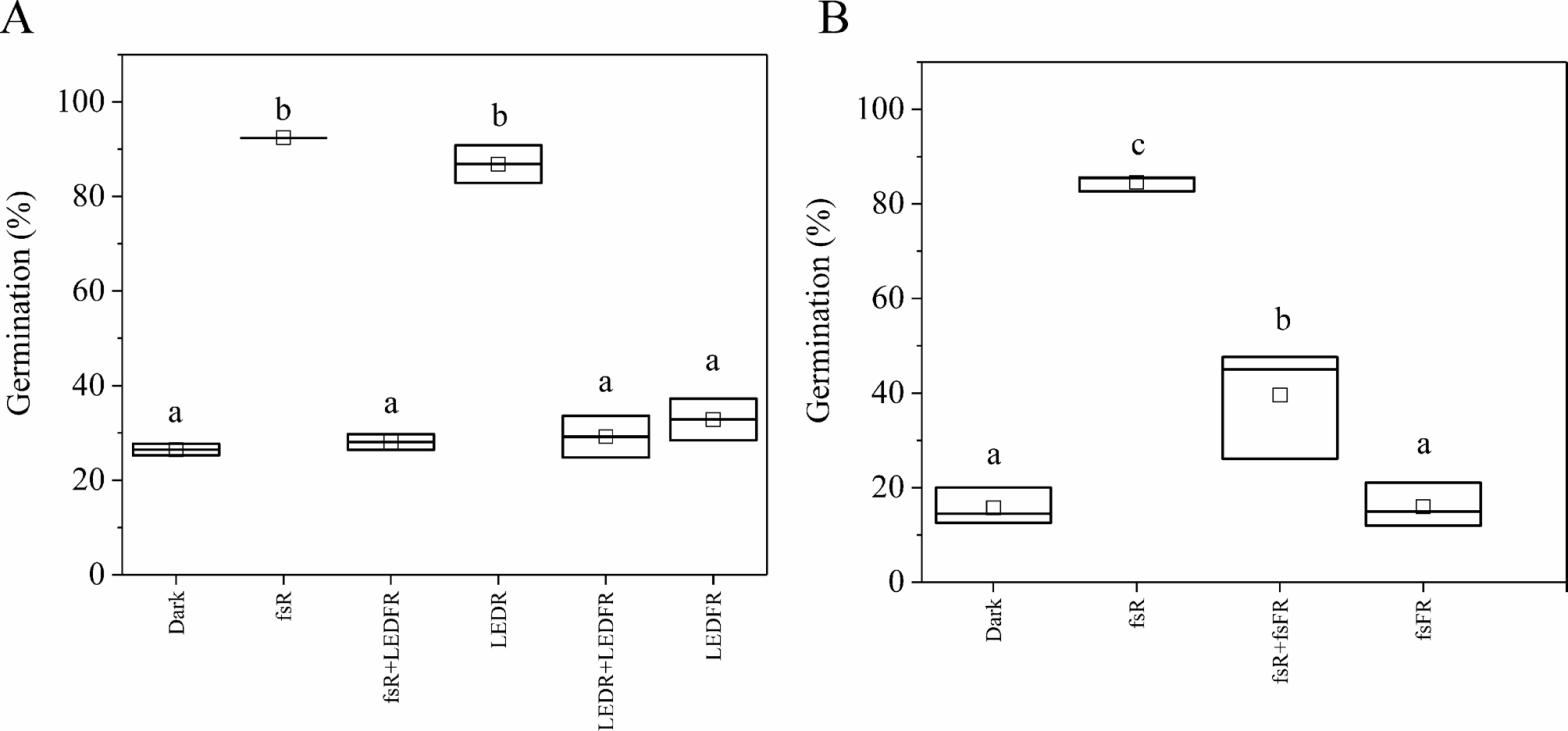
Germination is affected by ultrashort femtosecond R and FR pulses. Imbibed wild-type Arabidopsis seeds were pulse-irradiated with different R or FR light sources, or with R irradiation followed by FR irradiation (R + FR). The germination was determined after 72 h dark incubation. (**A**) The seeds were irradiated with R light at a fluence rate of 8.5 µmol m^– 2^ s^– 1^ (total fluence: 8.5 mmol m^– 2^), or with FR light at a fluence rate of 95.3 µmol m^– 2^ s^– 1^ (total fluence: 11.4 mmol m^– 2^) or with R and subsequently with FR light, using the following irradiation parameters: fsR Ti: Sa (100–120 µW, 1000 s); fsFR (1000–1100 µW, 120 s); LEDR (100 µW, 1000 s); LEDFR (1000 µW, 120 s). Non-irradiated control (Dark) was also included. The measurement was performed in two replicates with the total number of seeds per treatment group being *N* > 160 and *N* > 70 per replicates in a subgroup (mean ± SE; *n* = 2). The letters indicate significant differences of the means (*p* < 0.05 by ANOVA followed by a Tukey Test). (**B**) The seeds were irradiated with fsR Ti: Sa (80 µW, 1000 s) at a fluence rate of 6.8 µmol m^– 2^ s^– 1^ (total fluence of 6.8 mmol m^– 2^ ) or fsFR Ti: Sa (750 µW, 120 s) at a fluence rate 71.5 µmol m^– 2^ s^– 1^ (total fluence of 8.6 mmol m^– 2^). Non-irradiated control (Dark) was also included. The measurement was performed in three replicates with the total number of seeds per treatment group being *N* > 170 and *N* > 50 per replicates in a subgroup (mean ± SE; *n* = 3). The line bisecting each box represents the median value; the lower and upper edge of the box indicate the minimum and the maximum values, respectively. The empty square within each box indicate the mean value. The different letters indicate significant differences in the means (*p* < 0.05 by ANOVA followed by a Tukey Test).

To investigate whether ultrashort femtosecond light pulses can modulate germination responses we applied femtosecond R and FR sets of pulses on imbibed seeds. Figure 6A demonstrates that R light irradiation of 8500 µmol m^– 2^ fluence stimulates germination similarly, whether using the fsR Ti: Sa laser (92.4%) or the LEDR light source (86.8%). The germination promoting effect of R irradiation treatments can be completely reversed by a short (120 s, 11400 µmol m^– 2^ fluence) fsFR or LEDFR irradiation (fsR + LEDFR and LEDR + LEDFR, 28.1% and 29.2%, respectively), resulting in similar values to the dark control or solely LEDFR-treated seeds (26.5% and 32.9%, respectively). The germination rate of seedlings treated with FR light did not differ significantly from the dark control, indicating that our LEDFR irradiation cannot induce germination.

In the next experiment, we tested the effect of femtosecond far-red (fsFR) irradiation. The fsR irradiation promotes high germination (84.5%), which could be reversed by a subsequently applied fsFR treatment (fsR + fsFR, 39.6%). Interestingly, the reversion effect of the same duration (120 s) of fsFR after the fsR treatment (fsR + fsFR) was less pronounced than the effects of fsFR or fsR + LEDFR irradiations (Figs. 6A, B). This phenomenon can be partly attributed to the lower average power and fluence of the fsFR laser, inherent to the nature of this type of laser light source, despite the applied fluences of LEDFR and fsFR were in the comparable range (11.4 or 8.6 mmol m^– 2^, respectively).

### PhyB mediates germination triggered by femtosecond pulses

**Fig. 7:**
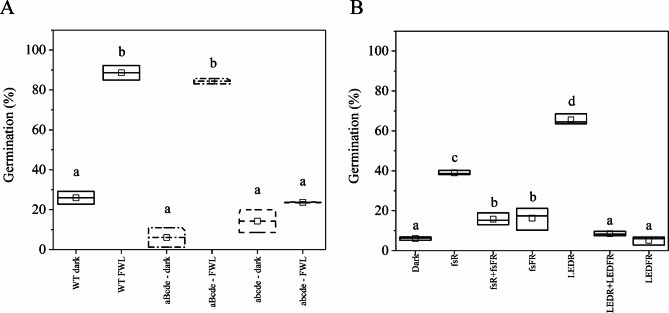
PhyB induced germination after FWL, R and fsR pulses. Imbibed Arabidopsis seeds were irradiated as indicated and kept at 22 °C for 72 h before germination rates were determined. (**A**) The WT, aBcde and abcde mutant seeds were irradiated with white light (FWL) or were kept in the dark for 72 h. (**B**) The aBcde mutant seeds were irradiated with fsR Ti: Sa, fsR + fsFR Ti: Sa, LEDR, LEDR + LEDFR and LEDFR irradiations or were kept in the dark for 72 h. The average powers and irradiation times were: fsR: 80 µW, 1000 s; fsFR: 1000 µW, 120 s; LEDR: 100 µW, 1000 s; LEDFR: 1000 µW, 120 s; resulting in the following fluences 6.8 mmol m^− 2^ (fsR), 11.4 mmol m^− 2^ (fsFR); 8.5 mmol m^− 2^ (LEDR) and 11.4 mmol m^− 2^ (LEDFR). Both measurements (panel A and B) were performed in three replicates with the total number of seeds per treatment group being *N* > 85 and *N* > 35 per replicates of a sub-group (WT, aBcde, abcde). The line bisecting each box represents the median value; the lower and upper edge of the box indicate the minimum and the maximum values, respectively. The empty square within each box indicate the mean value. Different letters above the boxes indicate significant differences of the means (*p* < 0.05 by ANOVA followed by a Tukey Test).

To investigate the molecular mechanisms underlying the observed germination response, we tested the efficiency of our experimental system on higher order phytochrome mutant lines. Wild-type Arabidopsis contains five (phyA-phyE), whereas the abcde mutant does not express functional phytochromes, and the aBcde line expresses only phyB. To have appropriate controls, we irradiated the seeds of these lines with continuous white light or kept them in the dark for 72 h. Figure 7A shows that wild-type and aBcde seeds germinated at significantly higher percentages under white light irradiation compared to those kept in the dark, while the germination rate of the FWL-irradiated abcde mutant seeds was much lower than that of the WT (23.7% vs. 88.6%). No significant difference was detected between the germination rate of the FWL- or dark-grown abcde seeds (23.7% vs. 14.3%). These results suggest that phyB-mediated germination is manifested in our experimental system, as it was observed under prolonged irradiation^54^.

We further investigated how the phyB photoreceptor alone can utilize short light pulses, generated by different light sources. We irradiated the seeds both with continuous and pulsed light, applying similar fluences for the R (6.8 mmol m^– 2^ of fsR and 8.5 mmol m^– 2^ of LEDR) and for the FR light (11.4 mmol m^– 2^ of fsFR or LEDFR) during single and R + FR combination treatments as well. The fsR and LEDR treatments induced significantly higher germination rates (39.0% and 65.6%, respectively) compared with dark incubation (6.2%). Additionally, a subsequently applied FR irradiation (fsR + fsFR or LEDR + LEDFR) reduced the germination rate to the corresponding dark levels (15.7% and 8.5%, respectively) (Fig. 7B).

Taken together, fsR and LEDR irradiation activates phyB, thereby inducing germination. In contrast, LEDFR and fsFR can revert Pfr to Pr or cannot induce Pfr formation. Consequently, phyB can be switched *on* and *off* not only by continuous but also by femtosecond pulsed irradiation.

### Post-imbibition femtosecond laser pulses can modify hypocotyl length

**Fig. 8:**
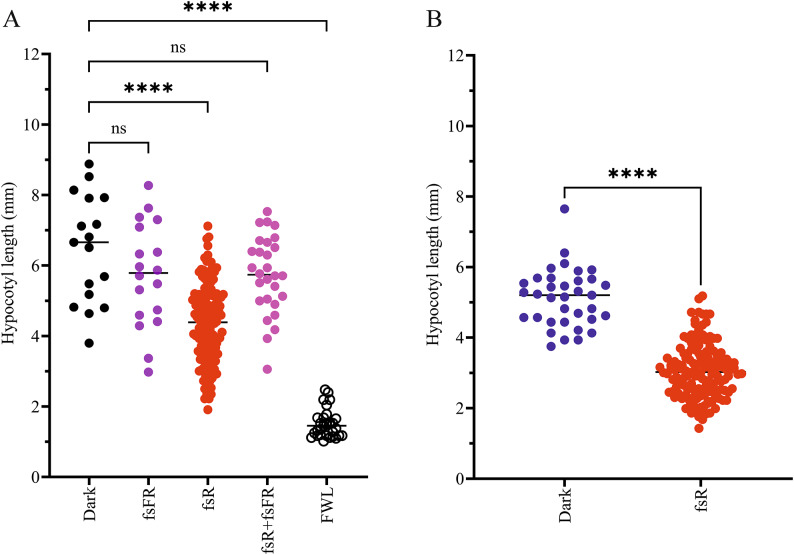
Elongation of hypocotyl is inhibited by fsR pulses. Wild type seeds were germinated in the dark with or without the indicated post-imbibition light treatments, except the FWL seeds that were kept under white light irradiation for 72 h at 22 °C. (**A**) Hypocotyl length of dark, fsFR, fsR, fsR + fsFR and FWL irradiated seedlings grown in 96-well plates. The applied fluence was 8.54 mmol m^− 2^ for fsR or 11.4 mmol m^− 2^ photon fluence for fsFR irradiation. (**B**) Hypocotyl length of fsR irradiated (8.5 mmol m^− 2^ photon fluence) and non-irradiated seedlings that were grown in full darkness in Petri dishes for 72 h. For both panels asterisks indicate significant differences of the means (**** *P* < 0.0001, ns *P* > 0.05 by ANOVA followed by a Tukey Test).

Along with investigating the effect of femtosecond light pulses on seed germination, we aimed to test whether these pulses influence the inhibition of hypocotyl elongation, a widely used indicator of photomorphogenesis^55^. In most studies, constant light is applied for several days, which is not feasible with femtosecond lasers. Therefore, we applied fsR pulses to imbibed seeds in the 10–1000 s time range, grew the seedlings in the dark for 72 h, and measured their hypocotyl length. Unfortunately, the number of seedlings was very low in the dark-grown and in the shortest (10 s) fsR irradiated groups due to the low germination rate. Therefore, we compared the hypocotyl length averages of the 30 s and 1000 s treatment groups and found that 1000 s fsR irradiation treatment inhibited hypocotyl elongation much more efficiently than the 30 s fsR treatment (Figure S4). To further examine this phenomenon, we applied fsR irradiation for 1000 s and found that it significantly inhibited hypocotyl elongation compared with the dark control (4.36 and 6.47 mm, respectively). Furthermore, fsFR irradiation cannot induce such an effect and reverts the effect of fsR, resulting in no significant difference between the average hypocotyl length of fsFR, fsR + fsFR and the dark sample (5.73 mm, 5.74 mm and 6.47 mm, respectively) (Fig. 8A). Not surprisingly, due to the presence of continuous illumination and other spectral components, the FWL treatment was more effective in the inhibition of hypocotyl elongation (1.51 mm). However, the applied short post-imbibition fsR pulses induced a significant, albeit partial response with only 100 ns of actual light exposure. Considering the suboptimal experimental setup for monitoring hypocotyl growth in the microtiter plate wells, we decided to observe the hypocotyl lengths of seedlings that are grown in water-filled Petri dishes (300 seeds in 10 mL) providing better air-circulation and more room for growth. This experiment corroborates our previous results and again clearly shows that a post-imbibition fsR pulse treatment can effectively inhibit hypocotyl elongation compared with the dark control (5.13 mm vs. 3.07 mm, Fig. 8B).

### Post-imbibition short laser irradiation does not cause adverse effects to adult plants

To assess whether the DLR treatment or the short fsR laser pulses have long-term effects on plant growth, we irradiated imbibed seeds, germinated and propagated them on soil, and measured various growth parameters. After 5 days of growth, we could detect only minor, insignificant differences in the hypocotyl length of seedlings evolved from irradiated or non-irradiated seeds (Figure S5). This suggests that the diurnal conditions (8 h light/16 h dark) in the growth chamber have a greater impact on seedling growth than the initial laser irradiation, particularly if we compare the results at the two sites. Next, we analyzed rosette parameters using PSI Plant Phenotyping systems. We measured rosette morphological parameters (plant dry mass, rosette area, perimeter, eccentricity, etc.) as well as key physiological indicators, including chlorophyll fluorescence (F_v_/F_m_), efficient quantum yield of photosystem II (Y(II)) and non-photochemical quenching (Y(NPQ)). Data collected from the two different locations using two phenotyping systems, indicated that neither DLR nor fsR laser treatments significantly affected the growth and development of adult Arabidopsis rosettes (Figures S6, S7, Table S1). We also conclude that laser irradiation of seeds does not damage plant growth and development.

## Discussion

### Method development

We developed a new irradiation technique based on seeds stirred in water suspension, which allows for their homogeneous irradiation with different light sources, even with narrow, collimated laser beams. The design of the custom-built setup (Fig. 3) facilitated the application of similar fluence rates of R and FR light, generated by fundamentally different light sources. This setup enabled the comparison of their biological effects through various plant phenotyping assays. Below are our main conclusions regarding the developed irradiation and plant propagation protocols:

(i) We used a 96-well microtiter plate reader^56,57^which provided reliable results with single endpoint imaging, enabling fast, high-throughput analysis of germination (Figs. 4, 5, 6 and 7).
(ii) We were able to distinguish between light treatments based on their ability to induce germination or inhibit hypocotyl elongation, a key phenotypic trait of seedling photomorphogenesis. Unlike most studies employing constant irradiation over hours and days, our laser-based approach provides shorter irradiation periods. After applying the light pulses, the seedlings were grown in the dark, which allowed us to compare our results with earlier studies. We observed that seedlings developed better when provided sufficient space, and ultrashort light pulses are able to induce seedling photomorphogenesis. Our results suggest that, together with germination rate determination, hypocotyl phenotyping, combined with the irradiation protocol, offers a reliable method to assess photoreceptor functions under these special light irradiation regimes (see later, Fig. 8).
(iii) We tested the growth of seedlings and adult plants on soil after the irradiation protocol. We concluded that seedlings could germinate when placed on soil immediately after laser irradiation, and when grown under day/night cycles, no significant differences were observed between plants developed from irradiated and non-irradiated seeds. Therefore, we concluded that the effect of post-imbibition seed irradiation is overshadowed by other parameters under these propagation conditions. Moreover, we observed that short, but intense laser irradiation of seeds does not have detrimental effects on plant development at later stages (Figures S5-S8, Table S1). This is an important observation, as numerous studies have highlighted the various harmful effects of short laser pulses, including photomechanical, chemical, and thermal impacts, when applied to living plant tissues^58^. The majority of reported harmful effects to living tissues were caused by focused beam irradiations with high peak intensities^59–62^. In contrast, in our experiments, the femtosecond laser beams were applied in a non-focused form with a relatively large diameter, making thermal and photochemical toxic effects highly unlikely.

### Short light pulses are sufficient to induce germination and seedling photomorphogenesis

It is well-established that Arabidopsis germination can be induced by light irradiation for a few minutes^41,63^. Here, we demonstrate that the intense irradiation of seeds with a cw R laser beam (DLR) for only 100 ms significantly induces germination. Furthermore, 10 s of such DLR irradiation results in the saturation of the germination response, leading to a germination rate similar to that induced by 72 h of white light irradiation (Fig. 4). This observation suggests that the brief light treatments generate a sufficient Pfr amount to effectively trigger the response, which is a key factor in light-induced germination, as previously proposed^13^. Collectively, these results indicate that phytochromes in the imbibed seeds are able to perceive even shorter and more intense radiation, than previously studied and can mediate physiological responses that are proportional to the fluence^63–66^.

Applying the same fluence and irradiation time for both incoherent LEDR and the coherent cw DLR light sources, we found that monochromatic DLR light is more efficient in germination induction than LEDR light with a broader spectrum (possible reasons are discussed in the next section). Even shorter, pulsed femtosecond light flashes can induce germination, although with lower efficiency than continuous light sources (Fig. 5). This difference can be due to the extremely compressed irradiation time: during the 1000-second-long fsR Ti: Sa laser irradiation treatment the effective light transmission time was only 100 ns, 1 million times (10^6^) shorter than the very effective 100 ms DLR treatment. Other possible explanations of this observation will be discussed later. We also note that if the generated pulses are not extremely short, and the seeds are irradiated in the second range, the law of linearity prevails: the same germination rate can be achieved by the combination of short irradiation time combined with high fluence rate or longer irradiation time together with lower fluence rate, provided the fluence is the same (Figs. 4 and 5). This observation matches the earlier observation on stem elongation^67^.

To further investigate the effects of short light pulses on other phenotypic traits, we examined whether these irradiation treatments of seeds could alter hypocotyl elongation. It is well-established that under R light, the inhibition of hypocotyl elongation requires the presence of phytochrome Pfr^68^. Our results demonstrate that the available Pfr in the seeds, irradiated with femtosecond R pulses, is sufficient to effectively inhibit hypocotyl growth in dark-grown seedlings. Similarly to germination, fsFR pulses can reverse the effect of fsR (Figs. 8 and S6). On the other hand, these changes are completely masked by later light treatments and cannot be observed in seedlings growing under light/dark cycles (Figure S5).

### Comparison of coherent and incoherent light sources

The fluence dependence of germination was measured for a variety of light sources. LEDR is an incoherent light source, which means the photons were absorbed by the molecules in random intervals, although absorption occurred in a very even manner throughout the duration of irradiation. On the other hand, continuous-wave lasers and ultrashort pulse lasers are coherent light sources, emitting light waves with the same frequency, wavelength, and phase. The DLR light source appears to be more efficient than LEDR, resulting in a higher germination rate at the same fluence rate (Figs. 5D, E). However, explaining the differing biological activity of these light sources is difficult. On the one hand, their emission spectra differ: DLR has a narrower emission range, providing irradiation in the optimal absorption range of the phytochromes, whereas the emission of LEDR is partly outside of that range (Fig. 1A). On the other hand, LEDR emits incoherent light, whereas DLR emits coherent radiation. Therefore, the correlation between the source types and the results can be further examined. For this, the preferred experimental setup would include optical elements in the light beam that can modulate coherence, allowing for the modification of this light parameter only.

### Phytochromes perceive femtosecond laser pulses

We noticed that whereas the absorption of femtosecond pulses by phytochromes and the subsequent conformational changes have been primarily investigated in bacterial phytochromes mostly in vitro using protein extracts^25–30^the impact of these pulses on phytochrome-mediated responses within living plants has not been studied. To examine whether ultrashort femtosecond light pulses can modulate germination responses, we applied femtosecond R and FR sets of pulses on imbibed seeds, establishing an experimental setup, similarly to the classical experimental arrangement used to activate/deactivate phytochromes^14^. We found that both fsR and fsFR pulses modulate seed germination and hypocotyl growth and mutant analysis indicated that phytochromes play a major role in these responses (Figs. 6B, 7 and 8). It is well-established from the examination of higher order phytochrome mutants that phytochromes (especially phyB) are key mediators of light pulse induced germination^46,54^. On the one hand, we found that the white light-irradiated phytochrome quintuple null mutant seeds do not germinate better than those grown in the dark, thus corroborating previous observations^69^ in a different Arabidopsis ecotype. On the other hand, our data clearly show that phyB alone is sufficient to induce germination at the same rate as all phytochromes in wild type seeds under FWL, and we found that it plays an important role under fsR irradiation (Fig. 7). This finding extends our limited knowledge of the physiological effects generated by femtosecond laser-light pulses. The most important aspect of femtosecond laser irradiation is the compression of the light pulses into ultrashort time periods. Since the Ti: Sa laser operates at 1 kHz repetition rates, one 100 femtosecond pulse is emitted every millisecond, thus the light/dark ratio of the irradiation treatment is 10^–13^ to 10^–3^, which is equal to the 1 to 10^10^ ratio (100 ns in 1000 s irradiation time). This ratio is the same as the proportion of 1 s to 300 years. Our results demonstrate that phyB molecules are capable of absorbing this radiation, which apart from being coherent, also contains a very high energy array of photons, and hence it drastically differs from continuous light sources. We also observed that not only phyB Pr absorbs fsR, but phyB Pfr absorbs fsFR, and both conformers transform these physical impacts into physiological responses (Figs. 7 and 8). Although similar in vivo studies, administering femtosecond laser pulses to plants have not been published, we can analyse our data based on in vitro studies. The relaxation of the light-sensitive chromophore of recombinant and native oat phytochrome A can be triggered by femtosecond pulses and these conformation changes are followed by further steps on the pico- and nanosecond range^25,30,70–72^. Similarly, the initial steps of the photoisomerization of Agrobacterium phytochrome have been determined to happen within 1 ps^73^ Similar ultrafast responses have been observed in light-absorbing proteins other than phytochromes from other organisms^74–76^indicating that biological systems, in general, are capable of absorbing femtosecond light pulses, and it is worthwhile to study the corresponding physiological responses. Collectively, these results, together with our findings indicate that femtosecond light pulses induce the same physiological mechanisms as traditional, incoherent cw pulses that were applied in the minute time range^41,54^.

To analyze the biological efficiency of different light treatments, we fitted sigmoid curves to normalized germination rate data, plotted as a function of log_10_(fluence). We found that the midpoints of the curves (EC50) are significantly higher for the fsR Ti: Sa laser compared with LEDR (1660 vs. 290 µmol m^-2^, Fig. 5D and F). This clearly indicates that LEDR irradiation is more effective in germination induction than fsR. The emission spectra of both light sources are centered at 660 nm, this range overlaps with the absorption spectrum maximum of phyB Pr. However, they are not monochromatic and contain light from the 625–690 (LEDR) or 630–670 (fsR Ti: Sa) nm ranges (Fig. 1A). Given the high similarity between these spectra, their lower LEDR EC50 value cannot be solely attributed to them. We assume that coherent light is not absorbed with lower efficiency (see the comparison of the DLR and LEDR light sources in the previous chapter); however, LEDR may be more efficient than ultrafast fsR pulses due to the generally lower light conversion efficiency at ultrashort light delivery times. We assume that the deviation from the Bunsen-Roscoe law of reciprocity^77^ observed here can be attributed to the highly limited duration of photon absorption. Similar reciprocity failure has been observed in different phytochrome-dependent responses generated by light pulses^78–80^. Under the 1 kHz Ti: Sa laser irradiation a 100 fs pulse was emitted every millisecond during the treatment interval. This pulse duration is close to the time scale required for the formation of the excited Pr S_1_ state. Considering the low photoconversion yield of Pr excitation (~ 15% Lumi-R formation) and the relaxation of the Meta intermediates back to Pr, the ground state is repopulated without re-excitation until the next femtosecond pulse arrives^30,34,35^, unlike with cw (LEDR or DLR) light sources. These differences in excitation dynamics may have, at least partially, contributed to the observed differences in efficiency between fsR and cw irradiation. With cw sources, the continuous illumination delivers fewer photons in any 100 fs duration than the 100 fs pulses of the fsR laser but the photons are continously present over time. Our data suggest that although the intense fsR pulses have high photon density and can excite the PHY Pr form to trigger the photoconversion to Pfr, they are insufficient to produce as many Pfr molecules as cw sources having the same fluence during the same total irradiation time due to their short duration and the characteristics of PHY photoconversion. This in vivo observation supports earlier in vitro data on PHY photoconversion^27,30,32,34,35,37,42^. Strikingly, irradiation with the 100 kHz fsR HR1 laser – despite having similar fluence rates but shorter pulse intervals (a 100 fs pulse every 10 µs) and delivering 100 times fewer photons per pulse compared to the Ti: Sa laser – resulted in germination rates similar to those observed under 1 kHz fsR irradiation from the Ti: Sa laser (Figure S3). We conclude that phytochrome light absorption is limited in time, at least under these extremely short light pulses.

In summary, we characterized phyB-specific photomorphogenic responses triggered by ultrashort femtosecond light pulses. These findings extend those in vitro studies that focused on the conformation changes of phytochromes induced by femtosecond pulses and demonstrate that these changes also occur *in planta*, leading to various physiological consequences. Further investigations are required to reveal the subtle details of this phenomenon.

## Conclusions

Our findings demonstrate that plants can perceive and respond to ultrafast changes in the physical properties of their surroundings, specifically to the temporal characteristics of light pulses. Here, we show that phytochrome photoreceptors can absorb photons, even when delivered within extremely short, femtosecond-duration pulses. Additionally, ultrashort (~ 100 femtosecond-long) light pulses can alter plant development, manifesting phenotypic differences even days after the irradiation. This demonstrates that the experimental system we developed is capable of in vivo monitoring of photoreceptor light sensing under extreme, non-natural light conditions. The fsR and fsFR ultrashort laser pulses, with precisely controlled pre-determined fixed time differences achieved through an optical delay line (ps-ns range) or other delay methods (µs-ms range), could also be very useful for further exploring the in vivo photoswitch nature of phytochrome molecules. Furthermore, this capability allows us to examine the formation of the active conformer at high temporal resolution.

In conclusion, seed germination and seedling photomorphogenesis can be induced by ultrashort, high-intensity light pulses, suggesting that plant photoreceptors do not require prolonged light exposures for activation. The fsR and fsFR pulses are indispensable for dissecting the intricate molecular mechanisms in vivo underlying phyB conformer changes. These pulses provide a high-resolution window into the fleeting, picosecond-to-nanosecond events that govern the initial light perception and conformational changes, which are foundational to downstream signaling and physiological responses like seed germination. Without this temporal resolution, many of the critical primary steps of the photoswitching would remain hidden. Therefore, we anticipate that future experiments, based on our presented results, may reveal the femtosecond-timescale of this photoswitch function *in planta*. This could be achieved by applying fsR and fsFR pulses with well-defined sequences and pre-set delay times, similar to those applied in pump-probe transient absorption measurements^30^.

Although our current findings do not have direct implication potential for agricultural and industrial applications, plant propagation under artificial light conditions is becoming increasingly important. This growing importance highlights the crucial need for our knowledge of plants’ physiological responses under artificial light irradiation and the ability to achieve precise light delivery and energy efficiency. As technology advances, femtosecond lasers have the potential to become a more valuable tool in plant biology, particularly in areas such as precision agriculture, environmental monitoring, and biotechnology. Delivering light with collimated femtosecond laser beams for different applications provides a very special tool for precise targeting both temporally and spacially. Future studies could uncover how different, economically important species respond to short, intense light treatments.

## Electronic supplementary material

Below is the link to the electronic supplementary material.

Supplementary Material 1

## Data Availability

Data is provided within the manuscript or supplementary information files.
