# Supplementary material for: A comparative study of femtosecond pulsed and continuous wave lasers on physiological responses through activation of phytochromes in seeds
Source: Sci Rep. 2025 Jul 23;15:26719. doi: 10.1038/s41598-025-11183-8 (PMC12287325; doi:10.1038/s41598-025-11183-8)
Supplement: Supplementary file 1 — Supplementary Material 1 [file 41598_2025_11183_MOESM1_ESM.pdf]

Supplementary information

A Comparative Study of Femtosecond Pulsed and Continuous Wave Lasers on Physiological Responses Through Activation of Phytochromes in Seeds

Csenger Márk Szabó[1,2+], Botond Bán[2+], Borbála Sinka[2], Bálint Tóth[1], Barnabás Gilicze[1], Imre Seres[1], János Bohus[1], Attila Ébert[1], Péter Borbély[3], Zsolt Gulyás[3], Gábor Galiba[3,4], Eva Darko[3], Miklós Hovári[5], Béla Hopp[2], Csaba Péter[6,7], Károly Mogyorósi[1], András Viczián[6*]*

[1]The Extreme Light Infrastructure ERIC | ALPS Facility, Wolfgang Sandner u. 3, H-6728 Szeged, Hungary

[2]Department of Optics and Quantum Electronics, University of Szeged, Dóm tér 9, H-6720 Szeged, Hungary

[3]Agricultural Institute, HUN-REN Centre for Agricultural Research, Brunszvik u. 2, H-2462 Martonvásár, Hungary

[4]Department of Agronomy, Hungarian University of Agricultural and Life Sciences, Georgikon Campus, Deák Ferenc u. 16, H-8360 Keszthely, Hungary

[5]Plant Stress and Phenomics Group, Institute of Plant Biology, HUN-REN Biological Research Centre, Temesvári krt. 62, H-6726 Szeged, Hungary.

[6]Laboratory of Photo and Chronobiology, Institute of Plant Biology, HUN-REN Biological Research Centre, Temesvári krt. 62, H-6726 Szeged, Hungary.

[7]Doctoral School of Biology, Faculty of Sciences and Informatics, University of Szeged, Közép fasor 52, H-6726 Szeged, Hungary

[+]These authors contributed equally.

*Corresponding authors

Table S1. Photosynthetic parameters determined during plant phenotyping.

(F_v/F_m): chlorophyll fluorescence; (Y(II): efficient quantum yield of photosystem II; (NPQ): non-photochemical quenching. Seed irradiation parameters are described in Figure S5. C: dark control; N: sample number; s.e.: standard error of the mean.

		C	N	s.e.	DLR	N	s.e.	P
**Site 1**	**Y(II)**	0.52933	25	0.02427	0.57143	24	0.00312	0.12207
	**Y(NPQ)**	0.13539	25	0.00434	0.13926	24	0.00120	0.61940
	**Fv/Fm**	0.75239	25	0.03152	0.79959	24	0.00486	0.20396
**Site 2**	**Y(II)**	0.66559	29	0.00334	0.67517	30	0.00298	0.14207
	**Y(NPQ)**	0.08926	29	0.00089	0.08543	30	0.00073	0.00239
	**Fv/Fm**	0.82250	29	0.00036	0.81268	30	0.00230	0.00920
		**C**	**N**	**s.e.**	**fsR HR1**	**N**	**s.e.**	**P**
**Site 1**	**Y(II)**	0.53184	29	0.00371	0.53525	28	0.00446	0.82514
	**Y(NPQ)**	0.17069	29	0.00465	0.16269	28	0.00392	0.40135
	**Fv/Fm**	0.79967	29	0.00334	0.80231	28	0.00328	0.85469

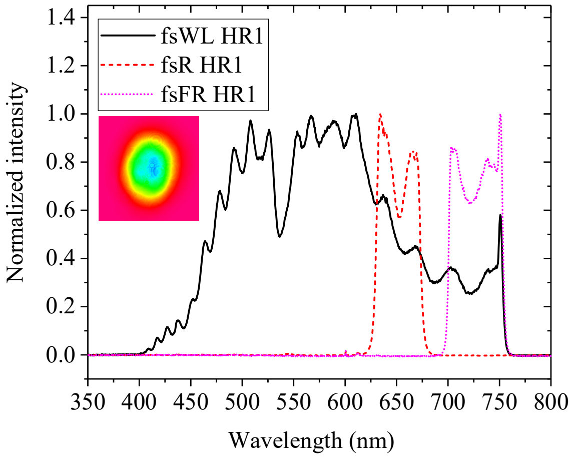

Figure S1. Emission spectra of femtosecond pulses generated by the HR1 laser.

Emission spectra of the fsWL (400–750 nm, with FES0750 short pass filter), fsR (FB650-40 band pass filter) and fsFR beam (with FEL700 long pass and FES0750 short pass filters) generated by the HR1 laser. The HR1 Gaussian beam profile is shown in the insert.

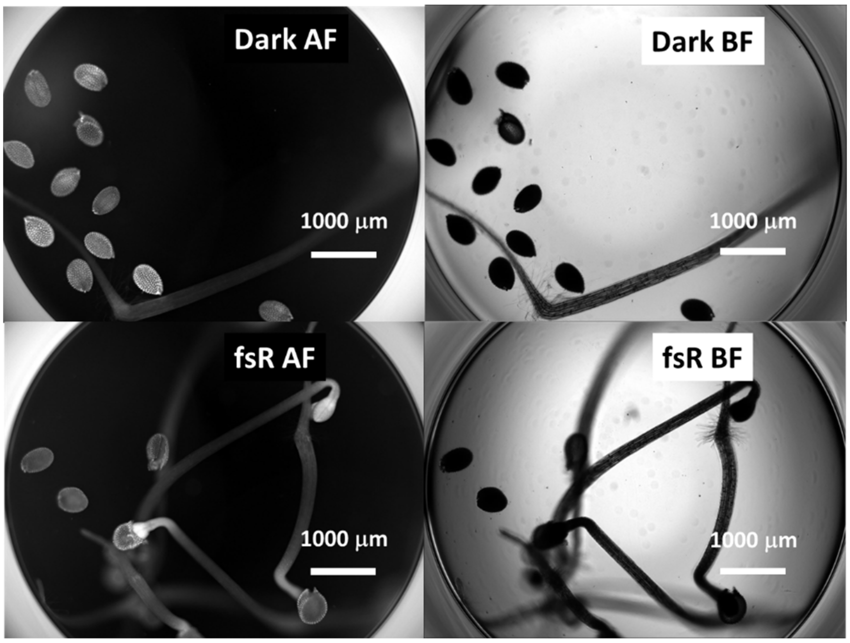

Figure S2. Example images generated by the EnSight Plate reader used for the assessment of germination assays.

Imbibed wild-type Arabidopsis seeds were kept in the dark (top row) or irradiated with pulsed femtosecond red laser for 1000 s (fsR Ti:Sa, 100 µW; 8500 µmol m^{-2}, bottom row). Autofluorescence (AF) and bright field (BF) images were taken by the EnSight plate reader after 72 h of dark growth.

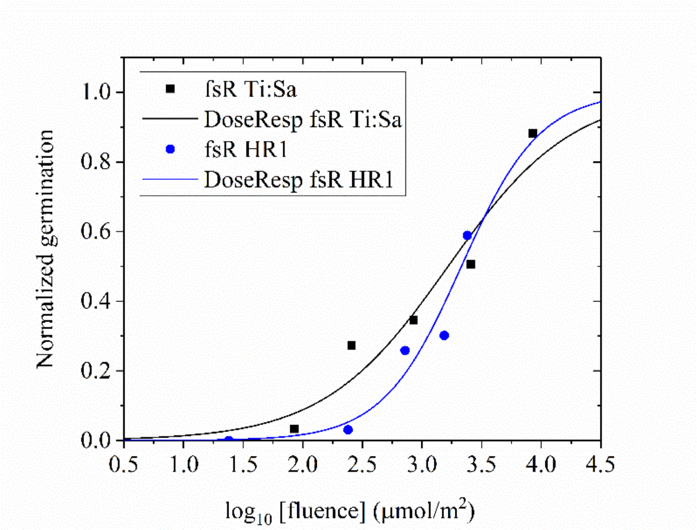

Figure S3. Fitting of the germination rate curve with a sigmoidal (dose response) function for fsR Ti:Sa and fsR HR1 treated seeds.

Measurement and calculations for fsR HR1 were performed as in Figure 5. The fsR Ti:Sa data is plotted from Figure 5.

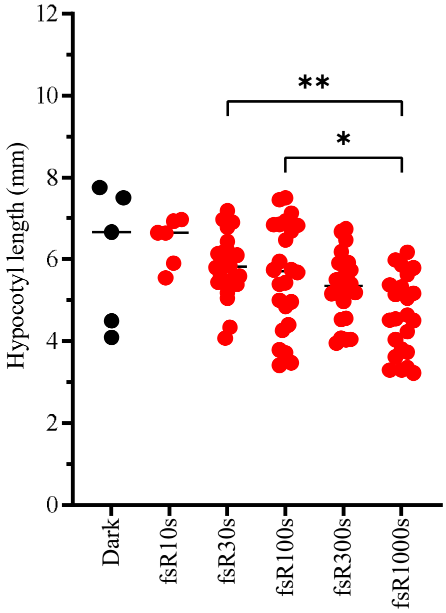

Figure S4. Femtosecond red pulses inhibit hypocotyl elongation.

Imbibed seeds were irradiated with fsR Ti:Sa laser pulses in the 10–1000 s time range or kept in the dark. After the irradiation treatments, the seedlings grew in the dark at 22 °C for 72 h, before their hypocotyl length was measured. The asterisks indicate significant differences of the means (**$p<0.01$ and *$p<0.05$) by ANOVA followed by Tukey Test).

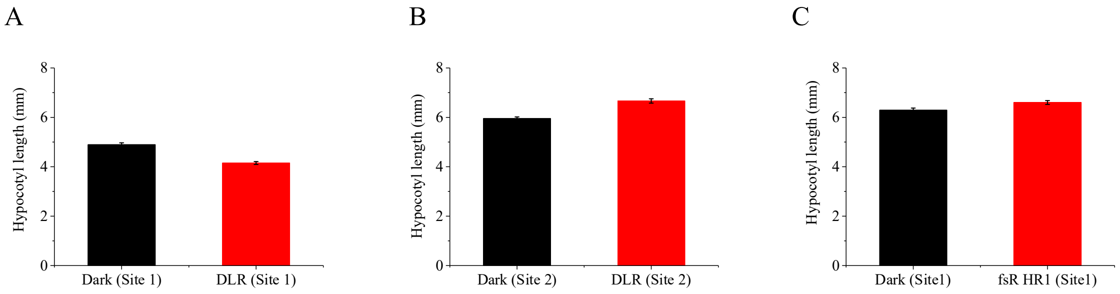

Figure S5. Hypocotyl length of 5-day-old seedlings grown on soil.

The hypocotyl length of seedlings grown on soil under dark/light cycles for 5 days after the seeds were irradiated for 30 s with DLR (A-B, 0.256 mmol m^{-2}) or with fsR HR1 (C, 0.256 mmol m^{-2}) light sources. The error bars depict the standard error of the mean.

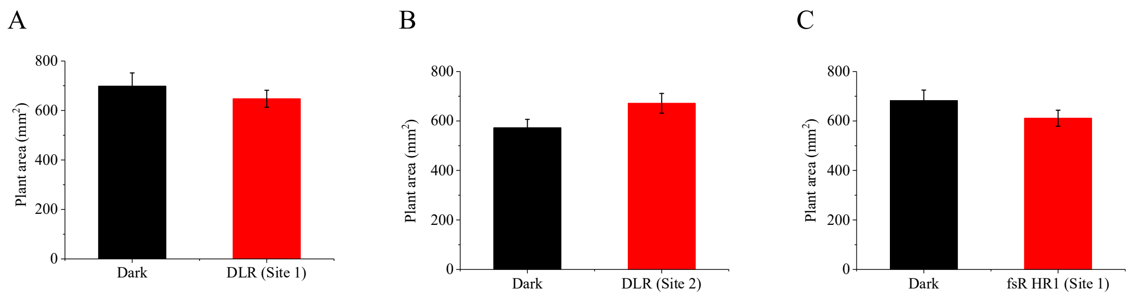

Figure S6. Plant area after 5 weeks of growth in soil.

Rosettes of 5-week-old plants developed from DLR (A-B) or from fsR HR1 (C) irradiated seeds and were measured using plant phenotyping instruments. Seed irradiation parameters are described in Figure S5. The error bars depict the standard error of the mean.

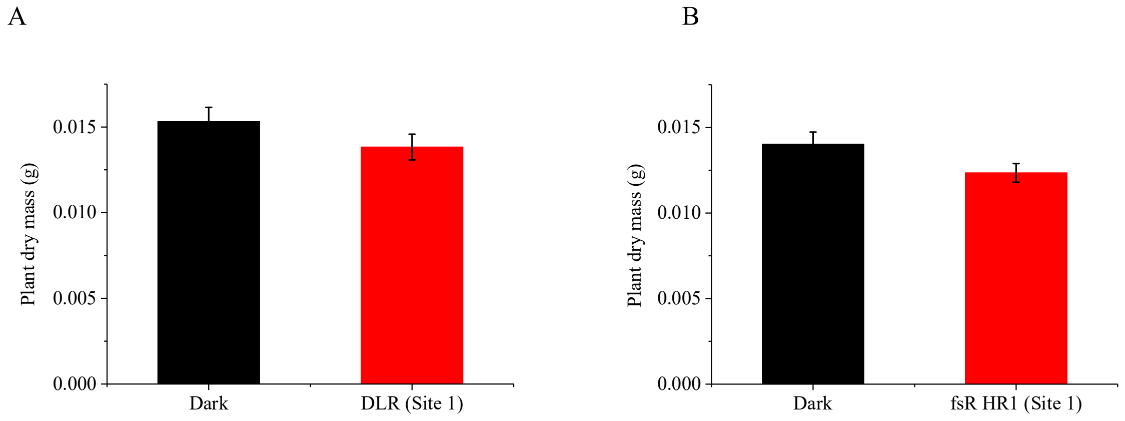

Figure S7. Plant dry mass of 5-week-old plants.

Plant dry mass was measured after 5 weeks of growth in soil under light/dark cycles. The seeds were irradiated with DLR (A) or with fsR HR1 (B) light sources as described in Figure S5. The error bars depict the standard error of the mean.

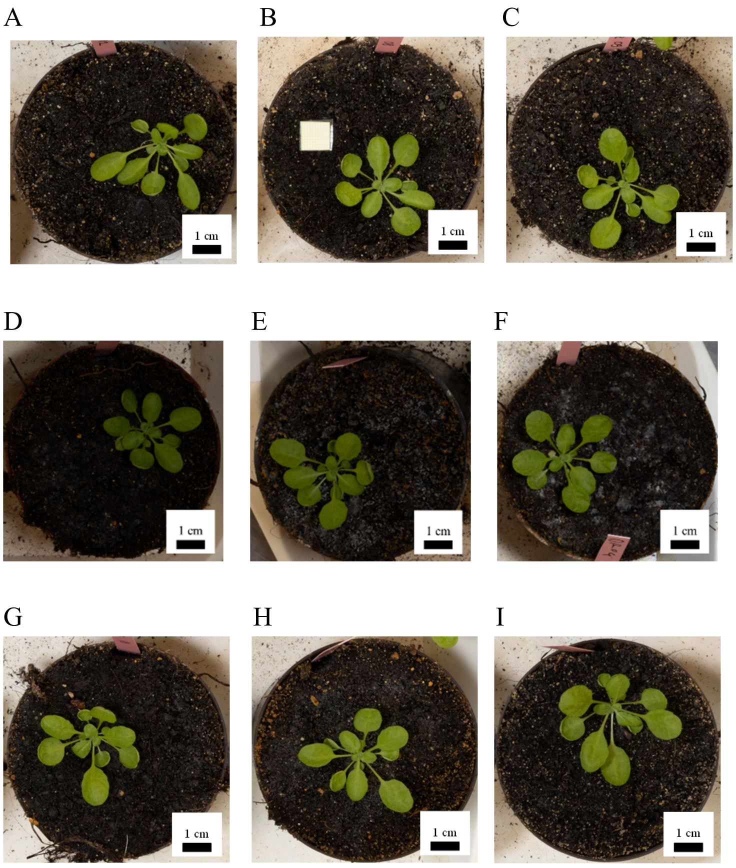

Figure S8. Representative images of adult plants.

5-week-old plants photographed at Site 1 at the time of phenotyping. After the imbibition, before being sowed directly on soil, the seeds were kept in the dark (A-C), were irradiated with DLR (D-F) or with fsR HR1 (G-I) as described in Figure S5.
